# Outcrossing in *Caenorhabditis elegans* increases in response to food limitation

**DOI:** 10.1002/ece3.11166

**Published:** 2024-03-20

**Authors:** Samuel P. Slowinski, Jennifer D. Gresham, Eric R. Cui, Katharine Haspel, Curtis M. Lively, Levi T. Morran

**Affiliations:** ^1^ Department of Biology Indiana University Bloomington Indiana USA; ^2^ Department Biology University of Maryland College Park Maryland USA; ^3^ Department of Biology Emory University Atlanta Georgia USA

**Keywords:** *C. elegans*, dauer, food restriction, mating system, offspring diversity, outcrossing, self‐fertilization, selfing, starvation, stress

## Abstract

Theory predicts that organisms should diversify their offspring when faced with a stressful environment. This prediction has received empirical support across diverse groups of organisms and stressors. For example, when encountered by *Caenorhabditis elegans* during early development, food limitation (a common environmental stressor) induces the nematodes to arrest in a developmental stage called dauer and to increase their propensity to outcross when they are subsequently provided with food and enabled to develop to maturity. Here we tested whether food limitation first encountered during late development/early adulthood can also induce increased outcrossing propensity in *C. elegans*. Previously well‐fed *C. elegans* increased their propensity to outcross when challenged with food limitation during the final larval stage of development and into early adulthood, relative to continuously well‐fed (control) nematodes. Our results thus support previous research demonstrating that the stress of food limitation can induce increased outcrossing propensity in *C. elegans*. Furthermore, our results expand on previous work by showing that food limitation can still increase outcrossing propensity even when it is not encountered until late development, and this can occur independently of the developmental and gene expression changes associated with dauer.

## Abstract 2

*Caenorhabditis elegans* increase their propensity to outcross in response to food limitation encountered during late development.

## 1 INTRODUCTION

Theoretical models have predicted that individuals that are poorly adapted to their environments (and have consequently experienced more stress) will benefit more than well‐adapted individuals from diversifying their offspring by increasing recombination rates (Agrawal et al., 2005) or by increasing biparental sexual reproduction (Hadany & Otto, 2007). Because inbreeding depression tends to increase under stress (e.g., Hayes et al., 2004) (reviewed in Armbruster & Reed, 2005), selection should favor increased rates of recombination and biparental sex in stressful environments. This leads to the prediction that organisms should diversify their offspring in response to indicators of low fitness, such as environmental stressors (Agrawal et al., 2005; Hadany & Otto, 2007). This prediction has received empirical support across a wide diversity of stressors and organisms. For example, exposure to parasites can cause snail hosts to increase their number of sexual partners (Soper et al., 2014), can cause *Daphnia* hosts to allocate more resources into male offspring production (at the expense of resource allocation into the production of asexual females) (Hite et al., 2017), and can cause fruit fly (Singh et al., 2015) and mosquito (Zilio et al., 2018) hosts to increase their production of recombinant offspring. In addition, *Strongyloides* nematode parasites reared in hosts with acquired immune protection are more likely to develop into sexual adults (Gemmill et al., 1997). *Drosophila melanogaster* raised at stressfully high temperatures (30°C) exhibit markedly higher recombination rates than control flies raised at a standard, non‐stressful, temperature (25°C) (Stern, 1926), and cold shock can also induce increased recombination in *D. melanogaster* (Zhong & Priest, 2011). Poorly defended (acyanogenic) *Trifolium repens* plants increase allocation to sexual reproduction in response to experimental exposure to herbivorous snails (Griffiths & Bonser, 2013). These examples suggest that parasites, herbivores, extreme temperatures, and, for parasites, their host's acquired immune system, are environmental stressors capable of inducing individual organisms to plastically diversify their offspring by increasing promiscuity, increasing allocation to biparental sexual reproduction (outcrossing), and/or elevating rates of recombination.

In the present study, we focused on food limitation as an environmental stressor. Food limitation is a common stressor experienced by diverse organisms. Several previous studies have shown that food limitation can induce organisms to diversify their offspring. For example, nutritional limitation increases bacterial competence to take up and recombine free strands of DNA from their environment (Jarmer et al., 2002; Redfield, 1993), induces meiosis in yeast (Mai & Breeden, 2000), increases recombination rates in *D. melanogaster* (Neel, 1941), and induces sexual reproduction and resting egg production in *Daphnia* (Kleiven et al., 1992; Koch et al., 2009).

The nematode *Caenorhabditis elegans* is a great model for assessing how the reproductive strategy of animals can change in response to environmental stressors (Alvarez et al., 2005; Morran et al., 2011; Morran, Cappy, et al., 2009; Plesnar‐Bielak et al., 2017; Slowinski et al., 2016, 2023) (reviewed in Anderson et al., 2010). *C. elegans* hermaphrodites are capable of multiple modes of reproduction: they produce both sperm and eggs and they can fertilize their eggs with their own sperm (self‐fertilization, or selfing) or with sperm acquired by mating with males (outcrossing). Relative to selfing, reproducing by outcrossing increases offspring genetic diversity, potentially enabling *C. elegans* to diversify their offspring by outcrossing in response to stressful environmental conditions.

Outcrossing in response to food limitation may be an adaptive strategy for *C. elegans* because, for *C. elegans*, food depletion likely serves a reliable cue that their offspring will disperse and encounter diverse habitats. *C. elegans* exhibit “boom and bust” population cycles (reviewed in Frézal & Félix, 2015). While food remains abundant, *C. elegans* will reproduce on their local food source with limited dispersal. However, once the food source is depleted, larva will enter an alternative developmental pathway and arrest at the second molt in a developmental stage called dauer. Dauers may leave their food‐depleted environment in search of new islands of resources, where they can complete development and reproduce (Felix & Duveau, 2012; Frézal & Félix, 2015). Hence, food limitation is predictive of offspring dispersal for *C. elegans*. Dispersal to diverse habitats is expected to be associated with low heritability of fitness (Williams, 1975), (i.e., under high dispersal conditions, variation across environments encountered by offspring, rather than parental genotype, explains a higher proportion of the variance in offspring success). Selection is predicted to favor parents that diversify their offspring when offspring are likely to encounter diverse and heterogeneous environments (Agrawal, 2009; Lenormand & Otto, 2000). Therefore, because food limitation is predictive of offspring dispersal, food limitation may be a good indicator of when outcrossing will be adaptive for *C. elegans*.

In order to investigate whether food limitation causes *C. elegans* to diversify their offspring, Morran, Cappy, et al. (2009) subjected *C. elegans* populations to food limitation and crowding, inducing the nematodes to arrest as dauers. As predicted, starved nematodes that had arrested as dauers exhibited an increased propensity to reproduce by outcrossing as opposed to self‐fertilization (after they had been provided with food and developed to sexual maturity) relative to well‐fed nematodes (control) that had not gone through the dauer stage.

While Morran, Cappy, et al. (2009) demonstrated that starvation during early development, and the corresponding induction of the dauer developmental stage, led to increased outcrossing propensity in *C. elegans* populations, it is unknown whether food limitation encountered at later stages of development can also induce increased outcrossing propensity in *C. elegans* that are no longer capable of becoming dauers. Increased outcrossing in *C. elegans* could be a general response to food limitation, or it could be a specific consequence of the extensive developmental, physiological, and gene expression changes (reviewed in Hu, 2007) associated with dauer. In the present study, we tested whether food limitation encountered late in development can induce increased outcrossing propensity. We raised nematodes on abundant food until they reached the final stage of larval development (L4), at which point we transferred a random sample of nematodes to a low‐food environment, and a random sample to a food‐abundant (control) environment. We assayed their propensity for outcrossing by pairing each individual hermaphrodite with a single male in their respective treatment environments, thus giving replicate hermaphrodites a standardized opportunity to mate. We measured male frequencies of the offspring produced on mating plates, and estimated outcrossing rates based on offspring male frequencies (Stewart & Phillips, 2002). We predicted that nematodes assayed in low‐food environments would exhibit an increased propensity to outcross relative to nematodes assayed in the control environment. The results were consistent with the prediction.

## 2 MATERIALS AND METHODS

### 2.1 Source of the experimental nematode populations

Our outcrossing propensity assays were run on one isogenic (inbred) *C. elegans* lab strain (CB4856; originally from Hawaii) and on one genetically diverse population called CW1‐30, which was derived by mutagenizing CB4856. In both assays, hermaphrodites were sampled from the mixed mating (wildtype) version of the strain, and males were drawn from a genetically similar obligate outcrossing version of the strain.

In the isogenic‐CB4856 assay, wildtype CB4856 hermaphrodites were paired with males from an isogenic obligately outcrossing strain of CB4856. The isogenic obligately outcrossing strain of CB4856 was created by introgressing the obligately outcrossing allele *fog‐2*(*q71*), which blocks the production of sperm in hermaphrodites and has no known effects on male phenotype (Schedl & Kimble, 1988), into a CB4856 genetic background.

The genetically diverse *C. elegans* populations CW1‐30 and F5‐0, which we will refer to as the mutagenized‐CB4856 assay, were derived as follows: Prior to our study, a mixed‐mating strain (designated PX382) was created by systematically inbreeding the strain CB4856 (Morran, Parmenter, et al., 2009). An obligately outcrossing strain (designated PX386) was created by systematically back‐crossing the obligate outcrossing allele, *fog‐2*(*q71*) into the genetic background of PX382 (Morran, Parmenter, et al., 2009). The mixed‐mating strain and the obligately outcrossing strain were mutagenized at 40 mM of ethyl methanesulfonate (EMS) for 4 h during three consecutive generations to introduce increased genetic variation (Morran et al., 2011). Following mutagenesis, the mixed‐mating strain and the obligately outcrossing strains were then passaged for 30 generations under standard laboratory conditions in the control treatment of another experiment (Morran et al., 2011), and the mixed‐mating and obligately outcrossing strains were both frozen. The strains were then thawed, and the mixed‐mating strain (designated CW1‐30) was used as the source of hermaphrodites in all assays, and the obligately outcrossing strain (designated F5‐0) was used as the source of males for our outcrossing propensity assays. A visual schematic representing how the mutagenized CB4856 strains CW1‐30 and F5‐0 were derived is presented in (see figure S1 in Slowinski et al., 2024).

### 2.2 Synchronizing the nematode populations for outcrossing propensity assays

Plates for rearing nematode populations were constructed by pouring 24 mL of autoclaved nematode growth medium (NGM) Lite (US Biological, Swampscott, MA) onto a 10 cm Petri dish. The NGM plates were seeded with a lawn of 60 μL of the *Escherichia coli* strain OP50, which was grown by inoculating 5 mL of liquid lysogenic broth (LB) with OP50, and then growing the OP50 culture for 24 h at 28°C, at which point the OP50 culture reached stationary phase. The seeded NGM plates were then grown overnight in a 28°C incubator before the nematodes were transferred onto the plates.

Prior to each assay, we synchronized the mixed mating and obligate outcrossing nematode populations by suspending populations in 1000 μL of liquid M9 (isotonic buffer solution), which was treated with 120 μL of 60% bleach solution to kill all the nematode life stages except the eggs (Stiernagle, 2006). Following exposure to the bleach solution, nematode eggs were washed with sterile deionized water and then continuously mixed in an M9 solution for 24 h in a tube rotator, during which period the eggs hatched into L1 larvae. All of the L1 larvae (estimated between 1000 and 2000 larvae) were transferred onto the seeded OP50 plates (one plate for mixed‐mating CB4856 worms and one plate for obligately outcrossing CB4856 worms in the isogenic CB4856 assay; one plate for mutagenized‐CB4856 worms and one plate for obligate outcrossing mutagenized‐CB4856 worms in the mutagenized CB4856 assay) where they grew at 20°C for 48 h until they matured to the L4 larval stage of development. Hence, all the nematodes in the study were provided with abundant food (on the plates seeded with 60 μL of OP50 culture) until reaching the L4 stage. They were then picked off of the abundant‐food plate and randomly selected for transfer onto low‐food or control mating plates. In the isogenic‐CB4856 assay, one hermaphrodite from the mixed‐mating isogenic‐CB4856 plate and one male from the obligately outcrossing isogenic‐CB4856 plate were transferred onto each (low‐food or control) mating plate. In the mutagenized‐CB4856 assay, one hermaphrodite from the mixed‐mating mutagenized‐CB4856 plate and one male from the obligately outcrossing mutagenized‐CB4856 plate were transferred onto each (low‐food or control) mating plate. Outcrossing propensity was assayed all at once in one big block in the isogenic‐CB4856 assay. The outcrossing propensity assay was repeated six times (six replicate blocks) in the mutagenized‐CB4856 assay.

### 2.3 Constructing mating plates for the outcrossing propensity assays

“Mating plates” are small agar plates that serve as arenas in which replicate hermaphrodites can experience a standardized opportunity to reproduce by outcrossing with a male or by self‐fertilization. Mating plates were constructed by pouring 4 mL of autoclaved NGM Lite (US Biological, Swampscott, MA) into a 3.5 cm Petri dish. Mating plates were seeded with 20 μL of undiluted stationary phase OP50 culture (control treatment), or with OP50 culture diluted by a factor of 10^−7^ for the low‐food treatment. The 20 μL of OP50 culture was spread across the mating plate using a sterile spreader and then incubated at 28°C for 24 h to allow the OP50 to grow prior to transferring nematodes onto the mating plates. The amount of OP50 that grew on the control‐treatment plates was well over the amount that could be consumed by the nematodes on the plates during the outcrossing propensity assays, hence the nematodes assayed in the control treatment experienced an unlimited food supply.

### 2.4 Assaying the outcrossing propensity of nematodes on mating plates

Our outcrossing propensity assays were adapted from (Bahrami & Zhang, 2013). In each assay, L4 hermaphrodites were randomly selected from our mixed‐mating population and individually picked onto mating plates, where each hermaphrodite was paired with an L4 male randomly selected from the genetically matched obligate outcrossing population. Importantly, L4 hermaphrodites have not yet reached sexual maturity, and cannot yet mate with males. Therefore, any outcrossing that we observed in our mating propensity assays has to have occurred during the assay period, after hermaphrodites and males were transferred to the mating plates. We will refer to the L4 hermaphrodites and males that we picked onto the mating plates as the “parents”, because they are the reproductive individuals whose outcrossing propensity we assayed. The parents were left together on each mating plate for 48 h at 20°C during which time the hermaphrodite on each plate developed to sexual maturity and had the opportunity to mate with the male and reproduce by outcrossing, or, alternatively, to reproduce by self‐fertilization. After 48 h, the parents were removed and their offspring, which were mostly still eggs or early larval stages, and which were easily distinguishable from the mature parents, were all transferred to a food‐abundant environment. Mating plates on which we could not find and remove the hermaphrodite mother (*n* = 0 in the isogenic‐CB4856 assay, *n* = 8 in the mutagenized‐CB4856 assay), or on which the mother had died during the 48‐h window of mating opportunity (*n* = 1 in the isogenic‐CB4856 assay, *n* = 1 in the mutagenized‐CB4856 assay), were excluded from our analyses. Food dilution treatment did not affect the probability that the mother was dead or missing after 48 h (isogenic‐CB4856 assay: *p* = .98; mutagenized‐CB4856 assay: *p* = .32). Mating plates on which the father had died during the 48‐h window of mating opportunity (*n* = 1 in the isogenic‐CB4856 assay; *n* = 0 in the mutagenized‐CB4856 assay) were also excluded. Food dilution treatment also did not affect the probability that the father was dead or missing after 48 h (isogenic‐CB4856; *p* = .98; no test run for the mutagenized CB4856 assay because no worms died in that assay).

Immediately after the parents were removed, we chunked each mating plate in its entirety onto a large NGM agar plate that had been seeded with stationary phase OP50, so that all the offspring from all treatments could complete their development in an environment with excess food. Forty‐eight hours after the parents were removed, we counted the male frequency of the offspring (which had matured into adults) on each mating plate by following a transect across the plate and scoring all the offspring as either hermaphrodites or males. After completing a transect across the plate, we moved up the plate to follow a new transect until the entire plate of offspring had been counted and all of the worms on the plate had been sexed. Mating plates on which we were unable to score the sex of at least 20 offspring, due to low offspring population sizes, were excluded from our analysis. In the mutagenized‐CB4856 assay eight out of 27 plates in the control and nine out of 25 plates in the 10^−7^ dilution treatment were removed from analyses due to insufficient number of offspring sexed. In the isogenic‐CB4856 assay there were sufficient numbers of offspring on all mating plates. Our results were not sensitive to the population size threshold that we used to determine which mating plates with low offspring population sizes to exclude; we got qualitatively similar results if we excluded mating plates with fewer than 30 offspring, 40 offspring, or 50 offspring sexed (data not shown). Food dilution treatment did not affect the total number of offspring that were scored on mating plates in the in the isogenic‐CB4856 assay (*F*_1_ = 0.62, *p* = .437; Table 1) or in the mutagenized‐CB4856 assay (ANOVA: *F*_2_ = 0.25, *p* = .78; Table 1). Note that the number of offspring sexed on mating plates should not be considered a measure of the lifetime reproductive success of the mother. Because hermaphrodites were picked onto mating plates prior to reproductive maturity and were removed from mating plates after 48 h, before they had completed egg laying, our offspring counts only represent the reproductive output of the early stage of egg laying. Linear models revealed that there was no significant relationship between the total offspring sexed and the outcrossing rate across mating plates the isogenic‐CB4856 assay (*F*_1_ = 3.5, *p* = .068) or in the mutagenized‐CB4856 assay (*F*_1_ = 0.018, *p* = .89).

**TABLE 1:** Median and range of total offspring sexed on mating plates from each food‐dilution treatment.

Worm strain	Dilution treatment	Median total offspring	Range total offspring	Number mating plates
Isogenic CB4856	Control	159.5	92–256	20
Isogenic CB4856	10^−7^	183.5	82–448	18
Mutagenized CB4856	Control	61	21–113	19
Mutagenized CB4856	10^−7^	59.5	20–114	16

*Note*: Data only includes mating plates that were included in the main analysis (i.e., mating plates that were not excluded due to insufficient offspring population size, or due to either parent being missing or dead at the end of the mating opportunity window).

### 2.5 Calculating outcrossing rates based on male frequencies

Because almost all of the offspring produced by selfing are hermaphrodites, whereas outcrossing produces a 1:1 ratio of males to hermaphrodites (Brenner, 1974), the outcrossing rate (i.e., the proportion of offspring produced by outcrossing) can be estimated based on male frequencies (Stewart & Phillips, 2002). Outcrossing rates of the parents on our mating plates were estimated as follows (Stewart & Phillips, 2002), assuming a nondisjunction rate of 0.0015 (Hodgkin & Doniach, 1997; Ward & Carrel, 1979):

$$\text{Outcrossing rate}=\left(\text{Male frequency}-\text{nondisjunction rate}\right)\times 2$$

### 2.6 Measuring the effect of food limitation on the production of males by nondisjunction of the X chromosome at meiosis

While outcrossing is the primary mechanism by which males are produced in *C. elegans* populations, rare males can also be produced by nondisjunction of the X chromosome at meiosis (Hodgkin, 1987). To confirm that any treatment differences that we observed in offspring male frequencies were due to the effects of food limitation on outcrossing propensity, and were not caused by food limitation induced changes in the frequency of nondisjunction males, we measured the rate of production of nondisjunction males in a control versus a low‐food environment. Previously well‐fed L4 hermaphrodites (from the isogenic‐CB4856 strain) were isolated on small Petri plates seeded with 200 μL *E. coli*. In the control treatment, LB was grown for 30 h at 28°C, and then used to seed the plates (*n* = 30) on which hermaphrodites were isolated. In the low‐food treatment, LB was grown for 30 h at 28°C, diluted by a factor of 10^−7^, and then used to seed the plates (*n* = 30) on which hermaphrodites were isolated. After 72 h, the hermaphrodite was removed from each assay plate, and the offspring were transferred to a plate with abundant (undiluted) *E. coli*, allowing offspring from both treatments to complete their development in a food‐abundant environment. The sex of all offspring on each plate was scored. Because the hermaphrodite mothers on the nondisjunction assay plates were isolated without access to males, all the offspring produced in this assay must be the product of self‐fertilization.

### 2.7 Statistical methods

#### 2.7.1 Outcrossing rate assays

We used a generalized linear model (GLM) to test whether food restriction treatment, nematode strain, and a treatment x strain interaction predicted outcrossing rates on mating plates. Because we found a significant main effect of treatment on outcrossing rates, we used Tukey's HSD post‐hoc tests to determine whether outcrossing rates differed significantly between treatments  within each nematode strain.

All *p*‐values reported are two‐tailed. All statistical tests were run using R version 4.1.3, and using R statistical package “emmeans” version 1.7.3 (Lenth, 2022).

#### 2.7.2 Nondisjunction rate assays

We used Welch Two‐Sample *t*‐tests to test whether the number of offspring sexed differed between the control and 10^−7^ food‐dilution treatment in each of the two non‐disjunction assays.

## 3 RESULTS

### 3.1 Outcrossing rates

**FIGURE 1:**
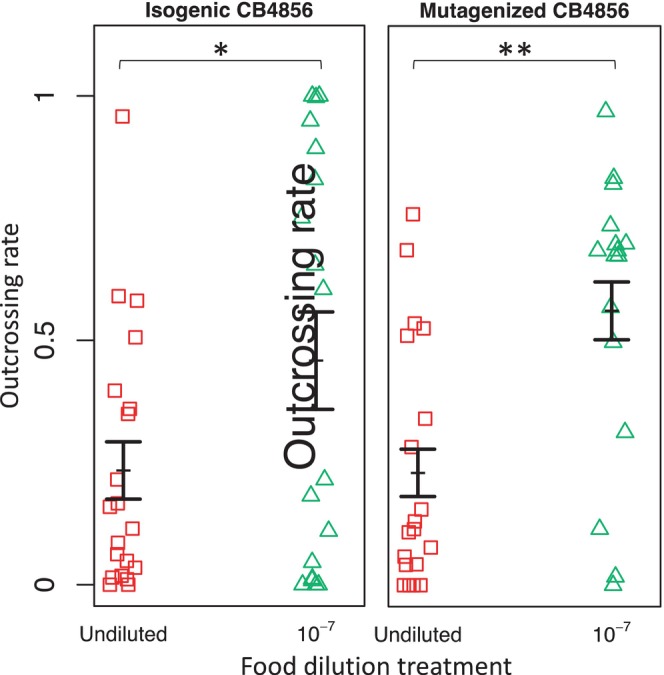
Outcrossing rates of hermaphrodites paired with males on mating plates with an undiluted *E. coli* food source (red squares) or with *E. coli* diluted by a factor of 10^−7^ (green triangles). Data pooled across replicate experimental runs. Each point represents the outcrossing rate on one mating plate. Left panel: hermaphrodites from the inbred mixed mating lab strain CB4856 paired with males from an obligately outcrossing strain of CB4856. Right panel: hermaphrodites from the mutagenized and genetically diverse mixed mating population CW1‐30 paired with males from the mutagenized and genetically diverse obligately outcrossing population F5 (both mutagenized populations derived from CB4856). Error bars represent ±1 standard error of the mean. *p< 0.05; **p < .01.

Food restriction significantly increased outcrossing rates on mating plates (deviance_1,71_ = 1.41, *p* < .001). There was no significant effect of nematode strain (deviance_1,70_ = 0.032, *p* = .57) on outcrossing rates. There was also no significant treatment x strain interaction on outcrossing rates (deviance_1,69_ = 0.04, *p* = .5; Figure 1).

Post hoc comparisons within each nematode strain revealed that the outcrossing rate was significantly higher in the low‐food environment than in the control environment for both the isogenic‐CB4856 strain (*t* ratio = −2.25, *p* = .028) and for the mutagenized‐CB4856 strain (*t* ratio = 3.1, *p* = .003).

### 3.2 Nondisjunction rate assay

**FIGURE 2:**
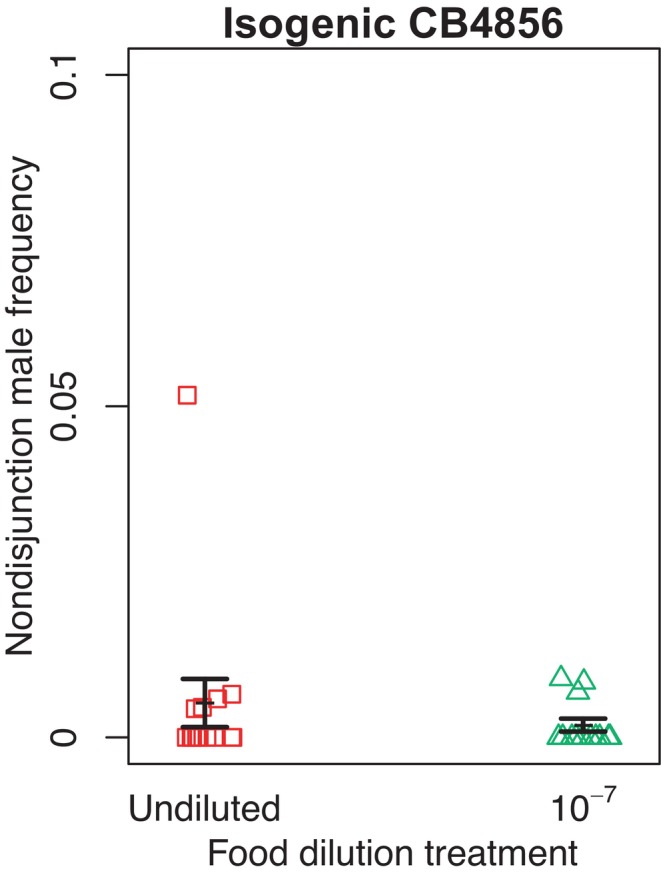
Frequency of nondisjunction (selfed) males produced by isogenic‐CB4856 *Caenorhabditis elegans* hermaphrodites assayed on control plates with an undiluted *E. coli* food source (red squares), or on plates with *E. coli* diluted by a factor of 10^−7^ (green triangles). Each point represents the frequency of male offspring on one assay plate. Error bars represent ±1 standard error of the mean.

In nondisjunction rate assay, we found no significant difference between the frequencies of nondisjunction males on control plates versus low‐food plates (*t*_14.9_ = 0.89, *p* = .39, Figure 2).

## 4 DISCUSSION

We found that food limitation, experienced during late development and early adulthood, can induce increased offspring male frequency in *C. elegans* that had been previously well‐fed until reaching the final (L4) stage of larval development. This increased offspring male frequency probably resulted from increased propensity of the parents to outcross, since increased outcrossing, rather than sexual conversion, has been previously shown to increase *C. elegans* male frequencies in response to food limitation (Morran, Cappy, et al., 2009). This same pattern of increased outcrossing in response to food limitation was observed in both an isogenic inbred *C. elegans* strain, as well as in a mutagenized and genetically diverse strain. Food dilution treatment did not affect the total number of offspring of either the isogenic or mutagenized strain. Furthermore, we found low rates of production of selfed nondisjunction, with no significant difference in nondisjunction rates in the food‐abundant control environment versus the low‐food environment. This suggests that the effect of food limitation on offspring male frequencies on mating plates was caused by food limitation induced changes in outcrossing propensity, rather than by any effect of food limitation on nondisjunction rates. Our results are consistent with theoretical predictions (Agrawal et al., 2005; Hadany & Otto, 2007) and with previous empirical research (Gemmill et al., 1997; Griffiths & Bonser, 2013; Hite et al., 2017; Singh et al., 2015; Soper et al., 2014; Stern, 1926; Zhong & Priest, 2011; Zilio et al., 2018) demonstrating that organisms plastically diversify their offspring in response to stressors in their environment. In particular, our results are consistent with previous empirical research demonstrating that starvation increases the propensity to outcross in *C. elegans* (Morran, Cappy, et al., 2009). Our results expand on this past research by demonstrating that increased outcrossing propensity in response to food limitation can still be induced in late stages of development and can occur independently of the developmental and gene expression changes associated with the dauer developmental pathway.

While our results demonstrate that food limitation can induce increased outcrossing propensity in *C. elegans*, outcrossing rates in natural (i.e., wild) *C. elegans* populations are estimated to be very low (Barriere & Felix, 2005, Barriere & Felix, 2007, reviewed in Felix & Braendle, 2010). Hence, even though food limitation is likely experienced frequently in natural *C. elegans* populations (reviewed in Frézal & Félix, 2015), the increased outcrossing propensity induced by food limitation appears to be insufficient to maintain high outcrossing rates in nature. Perhaps this is because other features of *C. elegans'* natural history limit outcrossing. For example, in nature, *C. elegans* subpopulations are typically founded by a small number of immigrating individuals that encounter a suitable food resource (Richaud et al., 2018). If male frequencies are low in nature, males will frequently be absent from the small founding groups that initiate new populations, resulting in no outcrossing opportunity even if outcrossing propensity is high.

The increased outcrossing propensity exhibited by *C. elegans* in response to food limitation may represent an adaptive strategy to diversify their offspring. Alternatively, *C. elegans* may outcross more in a low‐food environment simply as a non‐adaptive side effect of the effects of food availability on movement behaviors and activity levels. *C. elegans* hermaphrodites increase their roaming behavior when they are in a nutritionally poor environment (Dillon et al., 2016; McCloskey et al., 2017; Sawin et al., 2000; Shtonda & Avery, 2006), presumably in an effort to find better food resources. This increased roaming could increase the encounter rate between hermaphrodites and males, and opportunities for outcrossing. Furthermore, food deprivation reverses the response to CO_2_ from repulsion to attraction in worms that were raised at ambient CO_2_ levels (Baugh & Hu, 2020; Bretscher et al., 2008; Guillermin et al., 2017; Rengarajan et al., 2019). Because *C. elegans* are aerobic, consuming oxygen and producing CO_2_, this attraction to CO_2_ could also increase encounter rates in food‐deprived worms. We recommend that future research investigate the potential mechanisms by which food limitation could induce changes in outcrossing propensity in *C. elegans*. In particular, variation in the gene *npr‐1*, shown to be important for CO_2_ sensing (McGrath et al., 2009) in *C. elegans*, could be important in regulating outcrossing propensity plasticity. Future research should also investigate the fitness consequences of increased outcrossing in low‐food environments.

Our results demonstrate that food limitation at the fourth larval stage can induce increased outcrossing propensity in the isogenic lab strain CB4856 (originally from Hawaii) and in the mutagenized and genetically diverse experimental line CW1‐30 (derived from the strain CB4856). However, it is unclear whether other *C. elegans* strains would show a similar response. Morran, Cappy, et al. (2009) demonstrated that exposure to dauer generates increases in male frequency in the CB4856 (Hawaiian) and in the JU440 (French) *C. elegans* strains, but not in the N2 (British) strain, suggesting that effects of food limitation on outcrossing propensity may be strain specific. Future research should test whether the ability of food limitation to induce changes in outcrossing propensity varies across additional *C. elegans* strains. Furthermore, future research should also assess whether other types of environmental stressors, such as salinity, high temperature, environmental toxins, and parasite exposure can also induce increased outcrossing propensity in *C. elegans*. Finally, future research should assess whether food limitation affects outcrossing propensity by altering the phenotype of hermaphrodites, males, or both.

Overall, our results contribute to a growing body of work demonstrating that organisms can diversify their offspring in response to stressors in their environment. This stress‐induced change in reproductive strategy could help to explain why so many plants and animals reproduce sexually despite the costs, and how inter‐ and intra‐specific diversity is maintained in nature.

## AUTHOR CONTRIBUTIONS

**Samuel P. Slowinski:** Conceptualization (equal); data curation (equal); formal analysis (lead); funding acquisition (equal); investigation (equal); methodology (equal); supervision (equal); writing – original draft (lead); writing – review and editing (lead). **Jennifer D. Gresham:** Investigation (supporting); methodology (supporting); writing – review and editing (supporting). **Eric R. Cui:** Conceptualization (equal); funding acquisition (equal); investigation (equal); methodology (equal); writing – original draft (supporting). **Katharine Haspel:** Investigation (supporting). **Curtis M. Lively:** Conceptualization (equal); formal analysis (equal); supervision (lead); writing – review and editing (equal). **Levi T. Morran:** Conceptualization (lead); formal analysis (equal); supervision (equal); writing – original draft (equal); writing – review and editing (equal).

## FUNDING INFORMATION

This work was funded by the National Institute of Health Common Themes in Reproductive Diversity program for a Predoctoral Fellowship to SPS (grant number 2 T32 HD049336‐11A1), by an Indiana University Hutton Honor's College Summer Research Scholarship to EC, and by a National Institutes of Health: Institutional Research and Career Development Award (IRACDA) to JDG (grant number K12GM000680).

## CONFLICT OF INTEREST STATEMENT

The authors declare that they have no competing interests.

## Data Availability

Our data files and data analysis code files have been uploaded as supplementary documents. Our data and code will be uploaded and made publicly available upon acceptance of our manuscript.
