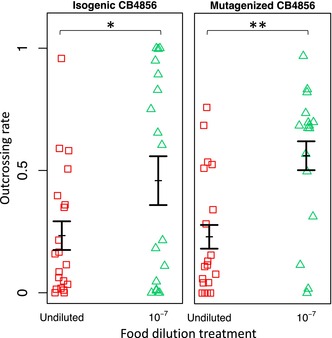# Correction to: Outcrossing in *Caenorhabditis elegans* increases in response to food limitation

**DOI:** 10.1002/ece3.11497

**Published:** 2024-06-03

Slowinski, S^1^, Gresham, J^1^, Cui, E, Haspel, K, Lively, C, Morran, L. Outcrossing in *Caenorhabditis elegans* increases in response to food limitation. 2024. Ecology and Evolution. doi: 10.1002/ece3.11166

^1^Equal author contribution

There is a formatting error in Figure 1, which was introduced by the publisher during the publication process after the authors submitted the manuscript. The Y‐axis in Figure 1 is duplicated, and the duplicated Y‐axis label blocks some data of Figure 1. The duplicated Y‐axis label has been removed in the corrected version.

We apologize for this error.

The correct Figure 1 is shown below.